# Physician payment methods: a focus on quality and cost control

**DOI:** 10.1186/s40463-014-0034-6

**Published:** 2014-08-03

**Authors:** Luke Rudmik, Dominika Wranik, Caroline Rudisill-Michaelsen

**Affiliations:** 1 Division of Otolaryngology – Head and Neck Surgery; Department of Surgery, University of Calgary, Foothills Medical Centre, South Tower suite 602, 1403 – 29th St. NW, Calgary, T2N 2 T9 AB Canada; 2 School of Public Administration, Faculty of Management, Dalhousie University, Halifax, NS Canada; 3 Department of Social Policy, London School of Economics, London, England UK; 4 LSE Health, London School of Economics, London, England UK

**Keywords:** Physician payments, Physician remuneration, Salary, Capitation, Fee for service, Pay for performance

## Abstract

With rising health care costs, governments must develop innovative methods to deliver efficient and equitable health care services. With physician remuneration being the third largest health care expense, the design of remuneration methods is a priority in health care policy. Otolaryngology-Head and Neck surgeons should have an understanding of the behavioural incentives associated with different physician payment methods. This article will outline the different physician payment methods with a focus on discussing the impact on quality of care and health care costs.

## Introduction

With rising health care (HC) costs and squeezed financing, governments must develop innovative methods to deliver efficient and equitable health care services. However, delivering efficient health care is inherently complex and relies on a stochastic process of evaluating disease probabilities and promoting desired behaviours by health care providers and patients.

A recent article by Wranik evaluated twenty-one Organization for Economic Co-operation and Development (OECD) countries concluding that policies targeting patient behaviour and physician remuneration were significant contributors to the efficiency of health care delivery [1]. Coupled with the fact that physician remuneration was the third largest health care expense in Canada in 2007, accounting for 13.4% of overall health care costs, the design of remuneration methods takes centre stage in health care policy.

It is commonly accepted that payment methods influence physician practice behaviour [2]. It is equally clear that non-financial incentives also play an important role in shaping behaviours. Insofar as remuneration methods contribute to behaviour, it is important to design them in such a way that the incentives they create are aligned with health care system goals.

This article aims to provide Otolaryngologists with a basic overview of the theory behind behavioural incentives associated with different physician payment methods and highlight the empirical evidence that supports or contradicts theoretical claims. We argue that evidence is not sufficiently strong which has served as the impetus behind the changes in physician remuneration introduced in Canada.

## Physician payment overview

The three pure physician payment methods include: fee-for-service (FFS), capitation, and salary. Since each have strengths and weaknesses, many jurisdictions have implemented blends of the three systems to combine the strengths and counteract the weaknesses. In addition, several jurisdictions have introduced various forms of pay-for-performance (P4P) systems in an attempt to improve HC quality through payment mechanisms.

When discussing a health care intervention, such as physician remuneration, defining the term ‘quality’ can be challenging since it often depends on perspective. A patient may define quality in terms of wait-list times, equity of access, continuity of care, or visit satisfaction. From a physician’s perspective, quality may refer to patient outcomes (i.e. disease free survival), professional autonomy, or practice satisfaction. From the health system perspective, quality may signify appropriateness of services provided and budget control. For the purposes of this article, quality will be discussed in terms of patient satisfaction, appropriateness of care, and continuity of care.

## Theory behind provider payment incentives

### Fee-for-service

The fee-for-service (FFS) system rewards the provider on a “per item of service provided” basis. Physician income is directly related to the number of health care services performed, creating an incentive to perform a high quantity of health care activities as financial risks associated with high volumes of activity sit with the payer. Since there is motivation to provide high amounts of services, it is in the physician’s interest to focus on patient satisfaction and therefore patient retention. The system also treats each patient equally creating equal incentives to accept high cost and/or low cost patients; a feature violated in a capitation or salary system. A FFS system is typically coupled with a provider-owned solo or group practice, which offers flexibility, autonomy and entrepreneurial opportunity.

A Swedish study by Forsberg et al. demonstrated that physicians are more aware of patient satisfaction when receiving FFS remuneration [3]. An American study by Sorbero et al. demonstrated that patients with stable chronic diseases were 36% more likely to switch from capitation-based providers to FFS-based providers. If switching providers is a measure of satisfaction, then FFS is associated with higher patient satisfaction for patients who require more health care services [4].

Devlin and Sarma propose that FFS remuneration tends to increase health care expenditure, primarily attributed to a greater quantity of care provided by physicians and higher administrative costs [5]. An important question then is whether the increased quantity of care provided is appropriate. When an appropriate quantity of care is provided, it can be viewed as ‘productivity’, however, an inappropriate quantity of care can be considered ‘supplier-induced demand (SID)’ [6]. Determining whether FFS increases productivity or SID is challenging. While some studies suggest that appropriateness of care is not affected by physician payment method [7],[8], several studies have demonstrated a risk of SID when compensation is tied to activities provided [9],[10]. Overall, FFS likely increases health care expenditure when evaluating patient visits, however, most health systems which utilize FFS remuneration control costs with other measures such as wait-lists or other rationing methods and/or implement user charges to curb unnecessary demand.

When the goals of a health care system are to increase patient satisfaction, quantity of care provided, and reduce the likelihood of selecting low risk patients (i.e. cream skimming), then FFS appears to be an excellent remuneration method. FFS is, however, likely to increase costs due to a greater quantity of services provided but disentangling whether volume increases are due to productivity gains or SID deserves further empirical scrutiny.

### Capitation

Capitation offers a “payment per patient per time period”. The incentive is to increase the clinical practice’s number of patients with no direct incentive to increase the quantity of health care activities provided. Therefore, the motivation is to accept relatively healthy patients, and reject (cream-skimming) or refer out (dumping) the relatively less healthy patients, since they will require a higher quantity of HC provided. For the payer, capitation is advantageous as providers face financial risks at the practice level.

A Norwegian study by Iversen and Luras demonstrated that when remuneration changed from FFS to capitation-based payment, physicians increased the referral of patients to private clinics for services that could have been provided by the referring physician [11]. In 2009, Glazier et al. compared capitation and FFS practice characteristics in Canada and demonstrated finding no differences in patient demographics, however, capitation was associated with more limited after-hours care, higher patient visits to the emergency department, and lower patient enrolment compared to the FFS cohort [12]. An American study by Hibbard et al. demonstrated that physicians with capitation-based remuneration were more motivated to ensure their patients are less reliant on medical organizations and promote patient self-care [13]. These results suggest that capitation-based remuneration may result in lower care continuity and reduced quantity of care. To prevent cream skimming, several capitation-based systems (UK NHS, other examples) utilize risk-adjusted capitations to compensate for higher risk patients.

Proponents of capitation argue that it controls costs by eliminating the incentive for SID and increasing disease prevention and health promotion [14]. The evidence suggests that capitation reduces quantity of care provided, and this may ultimately reduce overall health care expenditure. However, there would only be a cost-reduction to society if capitation remuneration reduced SID while still maintaining productivity. Furthermore, several recent studies suggest there may be no difference in disease prevention and health promotion between capitation and FFS [13],[15],[16]. Results of natural experiments with capitation in comparison to other payment methods do not solidly support expectations from theory; policy makers cannot claim to implement capitation systems due to supporting evidence. A risk-adjusted remuneration method is critical to maintain access equality when considering capitation-based payment.

### Salary

Salary is a fixed payment “per period of time” remuneration method. Therefore, payment is not dependent on the number of health care activities nor the number of patients. This payment method creates a stable, predictable income source for physicians, but also provides the incentive to reduce quantity of care. Salaries are an excellent method to recruit and retain physicians to under-populated or under-supplied regions, whereas FFS or capitation-based systems would inadequately reward physician efforts [17]. Salaries often have pre-negotiated services and work-hour stipulations built into contracts to maintain productivity and health care goals.

Proponents of salary-based physician remuneration state that it improves quality of care by increasing disease prevention, health promotion, and professional collaboration. A Canadian study by Battista and Spitzer demonstrated that salaried physicians performed more mammograms, fecal occult blood tests, and Pap-smears than FFS physicians [14]. A United Kingdom (UK) study by Gosden et al. evaluated behaviour in salaried primary care physicians (PCPs) compared to FFS or capitation-based PCPs. The results demonstrated that salaried PCPs had smaller patient lists, provided shorter consultations, prescribed less, and spent less time on administration. In salaried practices, quality was rated higher in seven out of thirteen clinical aspects compared to only two aspects for FFS/capitation practices [18]. However, two North American studies have demonstrated conflicting evidence, finding no difference in preventative care practices or self-help promotion between salaried and FFS physicians [13],[16].

Salary payment methods are believed to control costs by minimizing SID and promoting increased appropriateness of care. Furthermore, fixed payments tend to lower administrative costs to the health care system. Despite these cost advantages, there is a risk that salary may come with societal opportunity costs by reducing productivity and under-providing appropriate care.

### Blended remuneration

There is increasing interest in blended physician remuneration, which combines the advantages from each method while minimizing the potential for negative behavioural incentives [19]. A recent article by Wranik and Durier-Copp outlined some commonly utilized blended payment methods: 1) FFS combined with capitation, which allows the physician to bill FFS while receiving a small fee for each patient in the practice, 2) capitation system combined with a FFS element, where physicians receive a fee to cover pre-defined services for each patient in the practice, while services that fall outside can be billed FFS, and 3) salary system combined with FFS, where physicians receive a fixed lump fee for practicing and can bill FFS while receiving a percentage of the billings as further remuneration.

It is still early to determine the full effects of blended payment methods on general practice. Combining a capitation method with FFS may increase health promotion and disease prevention, while maintaining productivity and patient access equality. Lower-density or undersupplied regions may benefit from a salary method combined with FFS, which increases physician recruitment and retention, while providing a financial incentive to maintain productivity. Future research is required to fully elucidate the effects of blended payment methods on quality and cost.

### Pay-for-Performance (P4P)

With the objective to improve the quality of health care delivery as well as efficient delivery of care, pay-for-performance (P4P) both remunerates and measures physician performance based on achieving certain clinical targets at the patient population level and quality goals. Although P4P is appealing in theory, policy makers continue to face challenges identifying and implementing the ideal P4P structure that appropriately motivates physicians [20]. Defining suitable endpoints for payment, which are likely to be health processes and not outcomes presents an ongoing implementation challenge. There are also a number of unintended consequences that can emerge in a P4P system [21]. Physicians may reduce quality or limit quantity of care in sectors that are not incentivized to focus on those which are. The fundamental physician-patient relationship can also change as doctors aim to reach performance targets and patients’ preferences or clinical priorities may become collateral damage.

Conflicting evidence on P4P highlights the complexity of this remuneration method’s design. When evaluating P4P in a PCP practice setting, a UK study by Doran et al. demonstrated accelerated quality improvements during the initial roll-out phases and failed to demonstrate a detrimental effect on non-rewarded clinical care sectors [22]. Similarly, another UK study found short-term quality improvements for two of three rewarded areas with slowed improvements once targets were met [23]. When implementing a P4P program in a public health system (e.g. Canada, UK, Australia, New Zealand), rewards tend to be ‘bonus’ payments in addition to the regular income of the physicians, and therefore are bound to increase costs. This increase in cost must be balanced with the increase in quality.

## Canadian remuneration methods

In order to align behavioural incentives with HC objectives without contributing to excessive increases in HC costs, there is an interest in developing novel physician remuneration methods in Canada. In the early years of implementing alternate payment plans (APPs) for physicians in Canada, several stakeholders thought APPs were going to replace FFS. However, in a 2011 report published by the Canadian Institute for Health Information (CIHI), the implementation of APPs (such as salary or capitation) occurred by adding them to an existing FFS payment scheme. The addition of APPs to FFS increased by 3.6% suggesting that APPs are primarily being used to augment behaviours such as prevention and health promotion while keeping FFS to maintain volume of care [24],[25]. Table 1 outlines the different APPs currently implemented in Canada.

**Table 1:** **Alternate Payment Plans in Canada [**[25]**]**

Alternate Payment Plan	Description	% of total APPs in Canada	Provinces
Block Funding	Used by specialty groups in academic centres	22%	Ontario and Nova Scotia
Blended	Typically Salary plus FFS	16%	Predominantly in Quebec
Capitation	Predominantly in PCP practices	16%	Concentrated in Ontario
On-Call	On-call stipends in addition to FFS	12%	Common in most Provinces
Salary	Predominantly in rural areas	11%	Newfoundland and Labrador and Northwest Territories
Contract	Service contract related payments	11%	Concentrated in British Columbia
Sessional	Hourly payment for community physicians who work part time	8%	Most Provinces
Northern incentives	Working in rural northern communities	4%	Concentrated in Ontario and British Columbia

*FFS*, Fee for service; *PCP*, primary care physicians; *APP*, alternative payment plan.

The challenge is that the implementation of APPs in Canada has typically been introduced to augment specialties with lower-than-average FFS incomes or remunerate physicians in low population regions. This has effectively increased the cost of physician remuneration when shifting from a pure FFS method. For example, in British Columbia the cost of changing from FFS to APP service contracts in the emergency department resulted in a cost increase of $12.8 million for service contacts [26]. Another example are the Physicians in the Academic Health Services Sciences Centres program who received an additional $225 million in funding in addition to FFS payments [27]. Lastly, implementation of a Capitation-based APP in the Ontario Primary Health Care program would provide a capitation income of $328,000 per physician in comparison to the average gross income of the same practice size of $226,200 in FFS payments [28]. Therefore, current APP implementation appears to increase overall HC cost, however, it is important that the cost input be put into context of the quality of physician output. Policy makers and physicians need to consider the behavioural incentives associated with each payment method rather than focusing purely on the cost. Future studies are needed to evaluate the impact of APPs and P4P on the quality of care provided to patient using several different output metrics.

## Conclusion

The theory behind physician remuneration methods has been highlighted in many studies. We understand that in principle, FFS motivates quantity of care, while capitation motivates acceptance of healthier patients. Salaries offer stable incomes and allow physicians to focus their practice on patient needs, rather than on billable services. In theory, the blending of payment methods can combine the advantages of the pure methods. Where blending of methods is not sufficient, bonus pay-for-performance programs may be able to motivate the provision of targeted services. Policy makers need to consider not only the expected behavioural incentives associated with payment methods but also empirical investigation of these theoretical predictions.
